# Electrical Stimulation Enabled via Electrospun Piezoelectric Polymeric Nanofibers for Tissue Regeneration

**DOI:** 10.34133/2022/9896274

**Published:** 2022-08-02

**Authors:** Guangbo Xia, Beibei Song, Jian Fang

**Affiliations:** ^1^College of Textile and Clothing Engineering, Soochow University, Suzhou 215123, China; ^2^National Engineering Laboratory for Modern Silk, Soochow University, Suzhou 215123, China

## Abstract

Electrical stimulation has demonstrated great effectiveness in the modulation of cell fate *in vitro* and regeneration therapy *in vivo*. Conventionally, the employment of electrical signal comes with the electrodes, battery, and connectors in an invasive fashion. This tedious procedure and possible infection hinder the translation of electrical stimulation technologies in regenerative therapy. Given electromechanical coupling and flexibility, piezoelectric polymers can overcome these limitations as they can serve as a self-powered stimulator via scavenging mechanical force from the organism and external stimuli wirelessly. Wireless electrical cue mediated by electrospun piezoelectric polymeric nanofibers constitutes a promising paradigm allowing the generation of localized electrical stimulation both in a noninvasive manner and at cell level. Recently, numerous studies based on electrospun piezoelectric nanofibers have been carried out in electrically regenerative therapy. In this review, brief introduction of piezoelectric polymer and electrospinning technology is elucidated first. Afterward, we highlight the activating strategies (e.g., cell traction, physiological activity, and ultrasound) of piezoelectric stimulation and the interaction of piezoelectric cue with nonelectrically/electrically excitable cells in regeneration medicine. Then, quantitative comparison of the electrical stimulation effects using various activating strategies on specific cell behavior and various cell types is outlined. Followingly, this review explores the present challenges in electrospun nanofiber-based piezoelectric stimulation for regeneration therapy and summarizes the methodologies which may be contributed to future efforts in this field for the reality of this technology in the clinical scene. In the end, a summary of this review and future perspectives toward electrospun nanofiber-based piezoelectric stimulation in tissue regeneration are elucidated.

## 1. Introduction

The concept of “tissue engineering” was first proposed in the late 1980s to confront the limitation of transplantation as a result of mismatch and donor shortage [1]. In tissue engineering, scaffold matrices are employed to substitute damaged tissue for restoring, maintaining, or improving tissue function. Biomaterial scaffold, cells, and factors are three key components incorporated for the regeneration or repair of damaged tissues [2]. Scaffolds should mimic the structure and function of extracellular matrix (ECM) to support cell growth and provide physicochemical cues for cell development. Plenty of researches have demonstrated that the characteristics of scaffolds, e.g., surface topography [3], wettability [4], and mechanical stiffness [5], can effectively mediate cell behaviors. Similarly, electrical stimulation (ES) has also revealed the effectiveness in modulating cell activities, including cell morphology, migration, proliferation, and differentiation after the discovery of the endogenous electric field and transmembrane potential in biological tissue [6].

The effect of ES on tissue function has been well known since the 1960s when a low-level electric current affecting the bone formation of adult dog was illustrated [7]. The effectiveness of ES on tissue regeneration was confirmed in the late 1960s; it was found that direct current could be favorable for the wound healing of rabbit ears [8]. Many researches have demonstrated that applied ES allows the alternation of cellular components (e.g., ion channels, membrane-bound proteins, cytoskeleton, and organelles) [9]. Subsequently, cell behaviors and functions can be modulated [10]. For example, the employment of ES enables the accelerated growth of neurite and axon and induces neural differentiation from the embryonic stem cell [11]. Nevertheless, the effects of ES on cell function vary in different studies due to the differences of applied ES frequency, intensity and duration, and utilized electroactive material [12].

Conventionally, external electrodes are used to discharge on the wound area for direct ES [13–15]. Afterward, conductive scaffolds were supplementarily combined with electrodes to improve the quality of electrical cue-based tissue therapy [16]. The direct contact between electrode and biological tissue or culturing media may lead to infection and alternation of culture environment (temperature rise, pH variation, and generation of harmful byproducts). Thus, conductive coils were placed around the cell culture system to generate pulsed potential in a noninvasive manner, mimicking the natural potential transfer in the human body [17]. Despite the application of direct ES, the external power and conductive connector are still inevitable. Recent developments of smart material have allowed the exertion of power-free ES with or without external stimulus [18–20]. Typically, the incorporation of triboelectric or piezoelectric material enables the self-powered therapeutic ES via harnessing passive human biomechanical energy [21–23].

Piezoelectric effect refers to the phenomenon of converting mechanical force into electrical signals and vice versa [24]. Given electromechanical coupling [25], the piezoelectric effect attracts great interest in sensing [26–30], motion monitoring [31–34], and energy harvesting [35–41]. With the rapid development of piezoelectric materials and structures, they have aroused a considerable amount of attention as a self-powered electrical stimulator in tissue regeneration. Compared with inorganic piezoelectric materials, piezoelectric polymers have better biocompatibility and flexibility, offering an excellent platform for tissue regeneration [42, 43]. Kong et al. elucidated that ES generated on poly(vinylidene fluoride) (PVDF) film promotes calcium cations (Ca^2+^) influx through voltage-gated channels [44]. The rat bone-marrow-derived mesenchymal stem cells (rbMSCs) activated by piezoelectric stimulation on the PVDF film are inclined to exhibit a high level of cell density and neuron-like differentiation [3]. Apart from the modulation of proliferation and differentiation, the ES on the PVDF film also allows the protrusion and contraction morphology of rbMSCs [19].

The required mechanical force for the self-activation of the piezoelectric polymer can be scavenged from the random movement of organisms [45–47] or the interaction of the cell and scaffold [18, 19, 22] without the need of external power and electric wires. In addition, external stimuli, such as ultrasound [48–50] and magnetic fields [51–55], are capable of controllably activating the piezoelectric material in a wireless and noninvasive manner. In particular, fibrous piezoelectric polymers with a nanostructure are sensitive enough to achieve significant deformation through subtle stress caused by cell traction, thus generating considerable electrical signal for cell activity modulation [56]. Moreover, the nanofibrous web can mimic the biological function and microstructure of the collagen fibers (a natural piezoelectric material in ECM), acting as a medium of bioelectric signal transmission and communication between cells [57].

Electrospinning technology is generally used to fabricate continuous nanofibers as a result of simple manufacturing device, low cost, a wide variety of spinnable materials, controllable process, and so on [58]. Since the combination of electrostatic stretching and electrical polarization process which are necessary for piezoelectric crystal formation, the electrospinning process has attracted unprecedented research enthusiasm to one-step fabrication of fibrous polymeric piezoelectric structures [59–61]. Furthermore, the controllable surface pattern [62] and tunable porosity [63] of the nanofibrous web can be achieved through adjusting electrospinning parameters for providing a favorable cell growth environment. Recent researches have demonstrated encouraging biological effects (i.e., morphology modulation, proliferation, and the differentiation of stem cell) triggered by physiological mechanical energy or ultrasound-mediated electrospun piezoelectric nanofibers. Therefore, improving research enthusiasm has been witnessed in the indirect electrical stimulation realized via electrospun piezoelectric nanofibers at the cell and tissue level.

**Figure 1:**
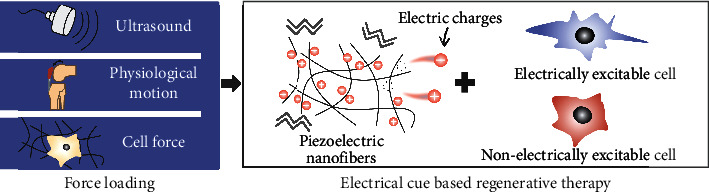
Activating strategies of fibrous piezoelectric polymer and the interaction between *in situ* electrical stimulation and cells for tissue regeneration.

This review begins with a brief illustration of the piezoelectric polymer, electrospinning technology, and the merits of the electrospun piezoelectric polymer nanofiber in tissue regeneration. Afterward, the activating strategies of piezoelectric nanofibers on cell growth, including cell traction, physiological activity, and ultrasound irradiation, are elucidated in detail, followed by the fundamental principle and the state-of-the-art applications concerning the interaction between piezoelectric stimulation and nonelectrically/electrically excitable cells (Figure 1). Next, the effects of the electrical stimulation supplied via various activating strategies on various cell types are quantitatively analyzed to evaluate which piezoelectric systems are more efficient on specific types of cells and on specific cell processes. In the end, we summarize the drawbacks of electrospun piezoelectric nanofibers in biological application and present solutions of optimizing electrospinning parameters, one-/two-dimensional hybrid composites, surface modification, and selected cells for preseeded cell-scaffold systems to propose a desirable electroactive scaffold for *in vitro* research and i*n vivo* clinical application on electrically stimulated-based therapy. From the activation of electroactive nanofibers, the molecular mechanism of cell response to piezoelectric stimulation, to the regulation of the cell with various cell lineages, this review is favorable to strengthen the comprehension of the interaction between piezoelectric nanofibers and cells and enable the sufficient exploitation of these paradigms from the decision of activating strategy to cell selecting. The challenges and solutions proposed upon piezoelectric nanofibers in regeneration therapy may partially reveal the puzzles and guide future efforts in wirelessly electrical stimulation therapy.

## 2. Piezoelectric Polymers and Electrospinning Technology

### 2.1. Piezoelectric Polymers

**Figure 2:**
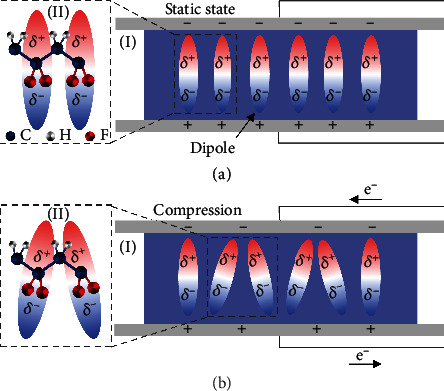
Dipole arrangement and electron transfer of piezoelectric polymer under (a) static and (b) compressed state. Note that II in (a) and (b) represents the chemical structure of PVDF.

The piezoelectric effect of piezoelectric polymers derives from the piezoelectric crystals which are formed from oriented dipoles (Figure 2(a), I). For the most common piezoelectric polymer of PVDF, the C-F bond constitutes the dipole unit due to polarity (Figure 2(a), II). Screening charges will be generated on the surface due to the overall polarization formed via aligned polymer chains (Figure 2(a), I). As the external force is loaded, aligned dipoles intend to randomly distribute (Figure 2(b), I) which slashes the overall polarization and in turn induces net electric charges in parallel electrodes (Figure 2(b), II). Due to the semicrystalline structure, the piezoelectric performance of piezoelectric polymers is inferior to that of inorganic piezoelectric materials [29, 64].

The flexibility of the polymer and the electromechanical coupling of the piezoelectric material makes the piezoelectric polymer attract unprecedented research enthusiasm in self-powered wearable sensors [32], tissue scaffold [65], and energy conversion [66]. Nevertheless, a high-intensity electric field combined with hot stretching is conventionally required for the fabrication of polymeric piezoelectric films [67]. The tedious and high-energy consumption process during conventional polymeric piezoelectric structure fabrication noticeably limits their industrial application.

### 2.2. Electrospinning Technology

**Figure 3:**
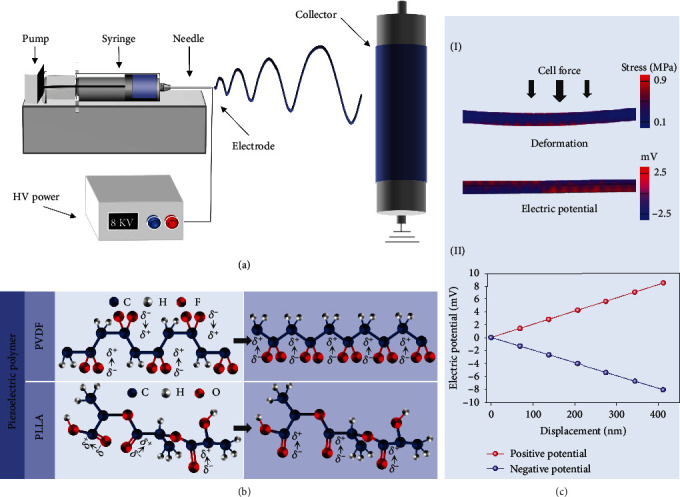
Electrospinning and piezoelectric nanofiber. (a) Illustrated setup of electrospinning. (b) Molecular structure changes of piezoelectric polymers during electrospinning. (c) Deformation and piezoelectric potential of single piezoelectric PVDF nanofiber under subtle force.

Electrospinning is of great importance for fabricating micro-/nanofibers due to its ease of fabrication, continuous process, low cost, and ability to formulate two-dimensional (2D) and three-dimensional (3D) structures [68]. A high-voltage power supply, syringe equipped with a needle, syringe pump, and grounded collector constitute a conventional electrospinning apparatus (Figure 3(a)). The polymer solution is loaded into a syringe and controlled through a syringe pump. A solution jet is initiated when the pump pushes the solution through the needle, and then, the jet flow is stretched into a nanosized fiber by the high voltage applied between the needle tip and the grounded collector. After solvent evaporation, nanofibers are finally collected on a conductive collector in the form of a nanofibrous web.

This simple, rapid, and continuous strategy can produce the nanofibers from a variety of materials, e.g., organic polymers and inorganic ceramics, as well as the composite of organic and inorganic materials [58]. In addition, the large specific surface area, high aspect ratio, and tunable porosity endowed by the electrospinning nanostructure are desirable for the application of filtration, water treatment, catalysis, and biological medicine [69].

### 2.3. Electrospun Piezoelectric Polymeric Nanofibers in Tissue Regeneration

Due to the strong electrostatic stretching on the solution jet, nonuniform domains in the polymer chain presumably diminish which leads to a higher net dipole moment. Additionally, the applied electrical field between the needle and collector is desirable for overall polarization of the piezoelectric material. As a result of the combination of electrostatic stretching and spontaneously electrical polarization, electrospinning technology has attracted great interest in the one-step fabrication of piezoelectric nanofibrous structure (Figure 3(b)) [70].

The flexibility of the electrospun piezoelectric nanofiber web is at a similar level to biological tissue, avoiding the tissue damage due to the mechanical mismatch between implanted piezoelectric material featuring other structures and biological tissues. The porous network endowed by the nanofiber assembly is desirable for the penetrating growth of cells and the transportation of metabolic wastes. In addition, piezoelectric nanofibers can mimic the biological function of piezoelectric collagen fibers in ECM which is a natural electromechanical conversion material for bioelectric signal transmission and communication between cells [57]. Piezoelectric polymeric nanofibers are more sensitive to the subtle force obtained from cell traction than other piezoelectric structures, indicating favorable potential as a self-powered electrical stimulator at the cell level (Figure 3(c)) [71]. Furthermore, the piezoelectric nanofibrous web with different structures and piezoelectric performance can be handily mediated through adjusting electrospinning parameters and controlling environment conditions, i.e., temperature and relative humidity (RH), exhibiting promising potential as a biological scaffold both supporting the living cell and supplying *in situ* electrical cue.

## 3. Activating Strategies of Electrospun Piezoelectric Polymeric Nanofibers

Mechanical loading on piezoelectric polymeric nanofibers is necessary for offering *in situ* ES on cultured cells. Apart from the strong mechanical energy achieved through ultrasound and physiological activities (i.e., joint bending and heartbeat), the subtle force from cell traction can also be detected as the mechanical force giver due to the sensitive structure of nanofibrous structures. The activating strategies of piezoelectric nanofibers for *in situ* electrical signal generation and cell behavior modulation are listed in Table 1.

### 3.1. Cell Traction Force

A series of cell activities, such as cell spreading and migration, occur during tissue regeneration. Therein, cell adhesion is the fundamental process [72], in which electromechanical signal communication occurs between the cell and scaffold or among cells when the extracellular environment changes. The three steps of cell contacting, flattening, and spreading are needed to form effective cell adhesion [73] where the stimulation of signal-regulated cell development is mediated [74–76]. When the cell intends to spread, the counterforce transformed from intracellular tension will be applied on the substrate due to the focal adhesion linking cell actin cytoskeleton to ECM. The counterforce generated in this process is referred to as cell traction force [77]. Conventionally, cell motion can trigger a force of about tens of nanonewtons (nN) at the cell adhesion site. This value is approximately tested utilizing specially patterned substrates (e.g., elastomers with micropillars). The distortion of the flexible microunit can be observed by the microscope, and the measurement of cell traction force can be further carried out on the basis of the linear relation between the distortion and force applied at each microunit [78]. In the case of the nanofibrous substrate, as shown in Figure 3(c), a single piezoelectric nanofiber is sensitive enough to respond to this subtle force (Figure 3(c), I) and induce the *in situ* electrical signal of several millivolts (Figure 3(c), II) [57]. Electrical cue from nanofibers can be sufficient to mediate the cell fate owing to the transmembrane potential for most cells ranging from -10 mV to -90 mV [79]. In addition, this ES with cell traction as a loop feedback signal can stimulate the cell accordingly, thereby avoiding the unfavorable effect of early electrical stimulation on cell spreading and adhesion [56]. Thus, cell traction is an ideal source of mechanical loading on piezoelectric polymeric nanofibers as a result of no external energy consumption and bidirectional electromechanical feedback. Besides, the interaction between piezoelectric nanofibers and cells proposes a promising therapy through providing ES at the cell level.

**Figure 4:**
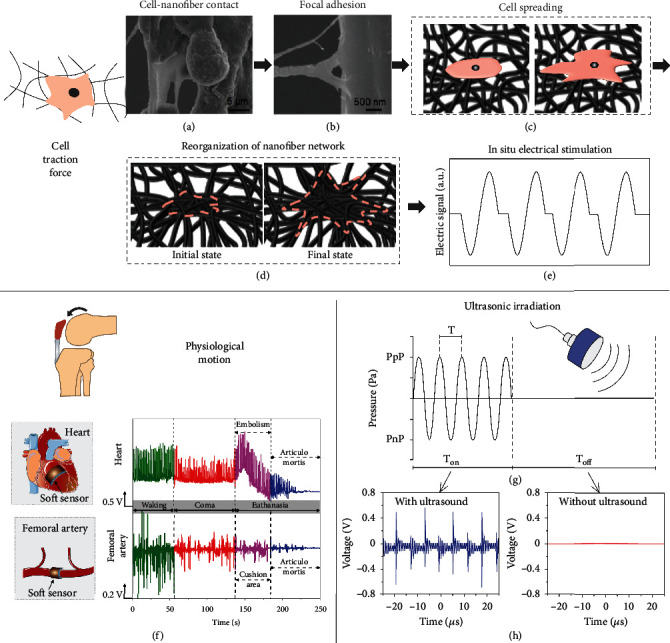
Electrical signal-inducing strategies of fibrous piezoelectric scaffold. Cell traction (a–e): (a) Cell-nanofiber contact, (b) focal adhesion, (c) cell spreading, (d) reorganization of nanofiber network, and (e) *in situ* electrical stimulation. (a, b) are reproduced with permission from Ref. [57], copyright 2021 *Science Advances*. Body activity and physiological environment: (f) Piezoelectric signals induced by heart and blood pulsing under different physiological states. (f) is reproduced with permission from Ref. [87], copyright 2019 *ACS Nano*. Ultrasonic irradiation (g, h): (g) wave shape of ultrasound; (h) electrical signal of piezoelectrical material irradiated by ultrasound. (g) is reproduced with permission from Ref. [48], copyright 2021 *ACS Nano*. (h) is reproduced with permission from Ref. [44], copyright 2021 *Advanced Science (Weinh)*.

When cells are seeded on a piezoelectric nanofibrous web, they first contact with nanofibers (Figure 4(a)). Then, focal adhesion is formed between the cells and nanofibers which is a bridge for transmitting cell motion to the deformation of piezoelectric nanofibers (Figure 4(b)) [57]. Otherwise, the cells exclusively slip on the surface instead of deforming nanofibers [56]. Next, the cell is inclined to spread around the nanofibrous web (Figure 4(c)) [80]. Due to the anchorage of focal adhesion, the arrangement of the nanofiber network will be reorganized in this process (Figure 4(d)) [5]. Finally, the deformed nanofiber generates *in situ* electrical stimulation in accordance with the strain level (Figure 4(e)) [81].

The extent of cell adhesion determines the mechanical force transmission from cell movement to piezoelectric nanofibers, therefore affecting the *in situ* stimulation on seeded cells. Surface property (e.g., wettability and surface zeta potential) of the scaffold and cell type are two main factors in the dominating cell adhesion condition. On the one hand, a hydrophilic surface is desirable for cell adhesion, and distinct tissue cells call for variable hydrophilicity. For instance, the hydrophilic surface is favorable for the spreading of the osteoblast and fibroblast on the culturing plate. Therein, the osteoblast prefers the super-hydrophilic surface (contact angel *θ* = 0°) while the fibroblast has maximum adhesion when contact angles are between 60° and 80°. In addition, the surface potential allows the changes of cell morphology. Both the negative and positive potentials on the piezoelectric platform lead to narrow and long cells which are capable of giving more adhesion sites [18]. It has been demonstrated that there is a linear relation between the amount of cell adhesion sites and induced piezoelectric electrical cues [19]. On the other hand, the cell type also influences the adhesion behavior. For instance, electrically excitable cells, such as neural and muscle cells, are more susceptible to electrical cues compared to nonelectrically excitable cells, therefore displaying more pseudopodia and synapses for cell adhesion. In particular, the spontaneous beating property makes the cardiomyocyte a potential candidate to activate the piezoelectric nanofibrous scaffold. Adadi et al. demonstrated that the number of 5 × 10^5^ cells was capable of activating the nanofibrous PVDF-TrFE web to generate a voltage signal of 7 × 10^−5^ V [81].

### 3.2. Physiological Activity

Daily body activity and physiological environment have also been reported as an *in vivo* mechanical loading giver [82, 83]. Mechanical force from the organism itself (e.g., joint bending and body motions during walking) is a good source to activate piezoelectric polymeric nanofibers *in vivo*. Piezoelectric nanofibrous webs have been implanted onto the tendon to scavenge mechanical force from joint bending [62, 84] or transferred into subcutaneous skin to sense motion force from walking and exercise [85]. It was demonstrated that a 6 *μ*A of current can be detected under cyclic pulling of the mouse leg when the PVDF-TrFE nanofibrous web was placed in the subcutaneous thigh region [85]. The electric signal variances were well consistent with the pause and pulling action. The coherent physiological activities, such as breathing and heart beating, also allow the *in vivo* activation of piezoelectric polymeric nanofibers. Azimi et al. demonstrated a heartbeat-driven voltage output of ~3.9 V on the nanofibrous ZnO/rGO/PVDF composite after implanting onto the heart of an adult female dog [86]. Furthermore, the mechanical energy can also be directly obtained from the micropressure of vascular walls. As shown in Figure 4(f), the core/shell PVDF/hydroxylamine hydrochloride (HHE) nanofibrous web was implanted onto the cardiovascular walls and femoral artery of an experimental pig. During the measurement, the micropressure change of vascular walls was mediated by regulating physiological states from wake to coma, and afterward to the euthanasia state. Under the wake state, the voltage output of PVDF/HHE nanofibers driven by cardiovascular walls was 1.02 ± 0.13 V while that induced through arterial pulsation was 0.52 ± 0.33 V. When the pig was in a coma state, the voltage output was decreased to 0.61 ± 0.24 V in response to cardiovascular walls and to 0.38 ± 0.25 V for blood flowing. The atrioventricular heart block and thrombus in the heart were observed in the euthanasia state due to the inhibition of excess anesthesia [87].

Condition simulation of *in vivo* mechanical loading is crucial in understanding the interaction between piezoelectric ES and tissue cells. A dynamic mechanical stimulus from a speaker (8 *Ω*, 1 W) [88] and linear motor [89] has been often incorporated to activate piezoelectric polymeric nanofibers *in vitro*. Variable frequency and intensity can be achieved to mimic the body motion through adjusting device parameters. For instance, Wang et al. demonstrated that a speaker with the sinusoidal signals featuring an amplitude of 4 V and frequency of 2 Hz could drive the PVDF-TrFE nanofibrous web to generate a maximum voltage output of -1.75 V [88].

### 3.3. Ultrasonic Irradiation

Despite the possibility of piezoelectric polymeric nanofibers being powered by the organism's motion, the stimulus controllability of electrical stimulators is rather undesirable [49]. Ultrasound is a wireless vibration stimulator which is extensively accepted in both diagnosis [90] and therapy [91, 92] owing to the features of high penetration depth, noninvasiveness, and safety to biological tissue (Figure 4(g)). Due to the acoustic cavitation effect of ultrasound irradiation, high localized pressure is generated at the interface of ultrasound-induced bubbles and liquid media [50], enabling the induction of localized electrical cues on piezoelectric nanofibers in a wireless manner [48].

It has been illustrated that low-frequency ultrasound has less absorption by the body, preventing heat dissipation from damaging local tissue [93]. Furthermore, low-intensity pulsed ultrasound is more favorable for tissue growth [94]. Hence, ultrasound with low frequency and intensity is generally exploited to deform piezoelectric nanofibers for generating *in situ* ES. The basic physical principle of this interaction is not yet clear, though a few models are proposed to describe the interaction between mechanical waves and piezoelectric nanoparticles [95–97]. Nevertheless, the simple quantitative relationship of ultrasound irradiation and the electrical output of the piezoelectric nanofibrous web has been measured [98]. For example, Das et al. illustrated an ~70 mV of peak voltage is measured on the PLLA nanofibrous web when the ultrasound with a frequency of 40 kHz is applied [71]. This piezoelectric output remains constant after being placed in cell media over the period of 26 days. In addition, the intensity of the piezoelectric signal irradiated through ultrasound depends on the intrinsic piezoelectric property of the nanofibrous PLLA scaffold mediated by the drum collector speed. It is more likely that ultrasound irradiation-assisted piezoelectric output is dominated by intrinsic piezoelectric performance rather than material structure. It was reported that a PVDF film with a piezoelectric coefficient (d_33_) of ~15 pc/N allows the generation of millivolt amplitude voltage similar to that of the PLLA nanofibrous web with a close piezoelectric coefficient [44]. As shown in Figure 4(h), the generated electrical signal under the ultrasound with a frequency of 80 kHz is consistent with the ultrasound irradiated output. Furthermore, there is a pulse period and pulse delay period during the ultrasound irradiation process; the voltage fluctuation period here is about 12.5 *μ*s which fits well with the ultrasound frequency. After polarization under a poling electric field of 70 kV/mm, the d_33_ of the PVDF film could reach ~60 pc/N enabling a piezoelectric output of the voltage level under ultrasound irradiation [49].

## 4. Interaction between Cells and Piezoelectric Electrical Stimulation

The nanofiber unit of the piezoelectric nanofibrous web will deform as the mechanical force from cell traction, physiological activity, and ultrasound irradiation is loaded. The charges or electric potential generated on strained piezoelectric nanofibers electrically stimulate the seeded cells. Cell membranes are electrically negative as a result of closed stacked assembly of lipids and membrane proteins [99, 100]. Thus, the electrostatic interaction between the piezoelectric electrical stimulation and cell may play a pivotal role in defining biological processes [101]. It is known that electrostatic attraction occurs in the case of opposite charges while electrostatic repulsion is engendered among same charges. Enhanced cell attachment and proliferation are generally elucidated on the positively charged substrates which is mediated via poling direction (determining the signal of surface electric potential/charges), as the cell membrane is negatively charged [102]. Nevertheless, the interaction of the negatively charged substrate and cell membrane will be inhibited since the positive ions in the biological fluid will be captured by the surface charges shielding this repulsive effect [100]. In this case, the lower poling degree (enabling the surface charge density or electric potential strength) of the piezoelectric material is more favorable for cell-substrate interaction. For example, Szewczyk et al. demonstrated that the nanofibrous PVDF with a -95 mV of surface potential contributes more to the adhesion and proliferation of the human osteoblast-like cell compared to that with a -173 mV of surface potential [102].

**Figure 5:**
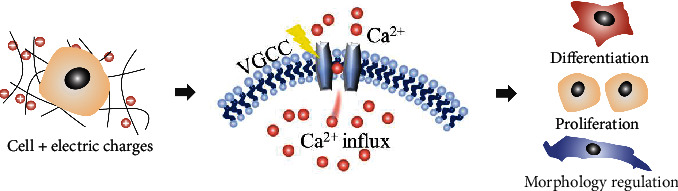
Principle of electric charge-modulated cell activities. Piezoelectric charges promote the influx of Ca^2+^; the increased Ca^2+^ on one side regulates cell proliferation and differentiation via interfering gene transcription and on the other side mediates cell morphology as a result of uneven distribution of Ca^2+^.

The plasma membrane depolarized via the mediation of membrane potential, receptor configuration, and receptor channels under piezoelectric electrical stimulation allows the reorganized distribution of extracellular and intracellular ions, ultimately regulating cell metabolism and development (Figure 5) [103]. Specifically, this electrical stimulation is capable of changing the local membrane potential which triggers the opening voltage-dependent channels (VGCCs) for high amplitudes of Ca^2+^ influx [22, 104]. Simultaneously, local electric potential can reorganize membrane receptors and open receptor channels, leading to the low-amplitude Ca^2+^ transients from the endoplasmic reticulum into the cytoplasm [22, 103]. These increased Ca^2+^ in the cytoplasm activate calcium-modulated proteins (calmodulin, CaM) [105] and finally promote the gene transcript to regulate cell proliferation and differentiation [106]. Moreover, the inhomogeneous distribution of electrical cue at the interface of cell and fibrous scaffold determines the increase of local Ca^2+^ concentration. The difference of intracellular Ca^2+^ distribution may cause actin depolymerization and consequently lead to cell contraction and protrusion which affiliates the cell migration [107]. Overall, the Ca^2+^ influx is the bridge for the piezoelectric charge to mediate cell differentiation, proliferation, and morphology, as illustrated in Figure 5.

### 4.1. Stimulation on Electrically Excitable Cells for Regeneration

**Figure 6:**
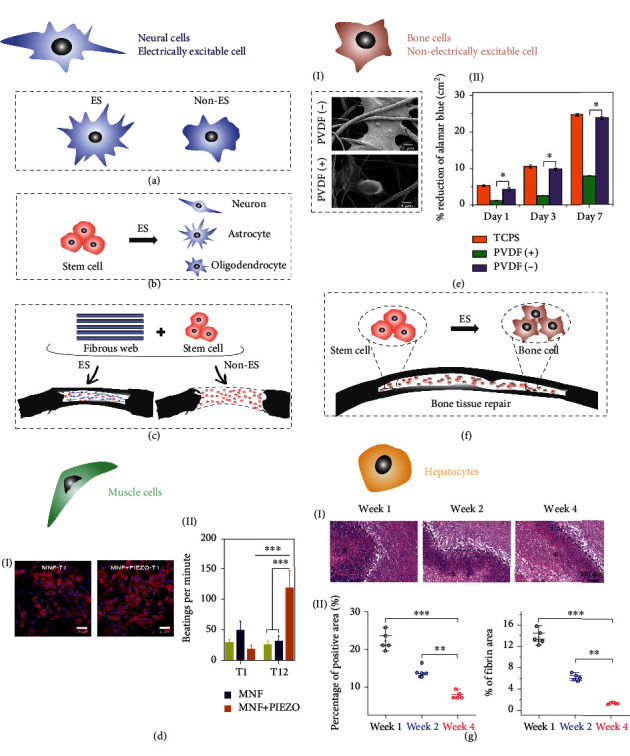
Electrical stimulation on electrically and nonelectrically excitable cells. (a–d) Electrically excitable cell: (a) cell morphology of neural cell under ES; (b) neural differentiation of stem cell under ES; (c) *in vivo* spinal cord repair of neural cell under ES; (d) morphology and maintenance of muscle cell. (d) is reproduced with permission from Ref. [115], copyright 2017 *Biomaterials*. (e–g) Nonelectrically excitable cell: (e) cell adhesion and proliferation of bone cell under ES; (f) bone differentiation from stem cell and *in vivo* bone repair under ES; (g) *in vivo* regeneration therapy of hepatocytes cell under ES. (e) is reproduced with permission from Ref. [102], copyright 2019 *ACS Biomaterials Science & Engineering*. (g) is reproduced with permission from Ref. [57], copyright 2021 *Science Advances*. Abbreviation: MNF: magnetic nanofilm.

The stereotyped response to the electric signal (e.g., action potential and cell contraction) defines the neurons and muscle cells as electrically excitable cells [48]. It is commonly accepted that ES determines neuronal excitability by depolarizing or hyperpolarizing the excitable cell membrane [108]. As shown in Figure 6(a), *in vitro* studies have demonstrated that more and longer cellular neurites are formed when dorsal root ganglion (DRG) neurons are electrically stimulated via PVDF–TrFE nanofibers [109]. Similarly, the cellular neurites of neural stem cells (NSCs) under the ES of PVDF nanofibers had an average length of ~91 *μ*m, which was much longer than the average length of ~40 *μ*m without ES [56].

Furthermore, the ES from piezoelectric polymeric nanofibers can also induce stem cell differentiation [110] which is a pivotal segment for regeneration medicine [111]. Liu et al. confirmed that the cell traction-driven ES on the PVDF nanofibrous web effectively induced NSC differentiation into neuron-like cells (Figure 6(b)). In comparison to chemically mediated differentiation, the neuron-glial interface induced by ES gives rise to strengthened interactions among cellular components, bringing desirable neural connectivity and functionality [112]. It is worthy to note that the piezoelectric nanofibrous PLLA web displays orthogonal and shear piezoelectricity, and the ES from orthogonal and shear directions of nanofibrous PLLA is responsible for the specific differentiation of NSCs into neurogenesis and osteogenesis, respectively [113]. *In vivo* experimental evidence indicates the capacity of the above-mentioned stimulation paradigm to help nerve tissue repair (Figure 6(c)). For instance, Lee et al., elucidated a PVDF-TrFE conduit fabricated by rolling the aligned PVDF-TrFE nanofiber web. After transplanting the conduit into the transection of the spinal cord, it can effectively promote peripheral nerve repair after 3 weeks [114].

It has been widely verified that piezoelectric cue-induced stem cell differentiation is always accompanied by the transient shift of Ca^2+^ from extracellular to intracellular, indicating the effect of the Ca^2+^ influx on differentiation [56, 115]. In addition, the piezoelectric potential generated on the piezoelectric nanofibrous web can drive iron ion (Fe^3+^) release, and the synergistic effect of the electrical cue-driven Ca^2+^ and Fe^3+^ of FeOOH/PVDF nanofibers promotes the differentiation of rat bone-marrow-derived mesenchymal stem cells (rBMSCs) into neurons without any neural-inducing factors [50].

In comparison with neural cells, only a few researches focus on the piezoelectric ES of polymeric nanofibers on muscle cells. Adadi et al. demonstrated that the ES from the nanofibrous PVDF-TrFE web differentiated human induced pluripotent stem cells (hiPSCs) into cardiomyocytes (CM) and then made it mature over the period of 40 days [81]. In this work, the PVDF-TrFE nanofibrous web simultaneously served as a biological scaffold and sensor to measure contractile function, indicating a potential candidate for disease modeling or cardiotoxicity studies in cardiac tissue. Besides, PVDF-TrFE nanofibers also provided *in situ* ES to elongate CM morphology (Figure 6(d), I) and preserved CM contractility for at least 12 days (Figure 6(d), II) [115].

### 4.2. Stimulation on Nonelectrically Excitable Cell for Regeneration

Electrically responsive action potential and contraction make neural and muscle cells defined as electrically excitable cells. Nevertheless, many other cell types, such as osteoblasts, chondroblasts, fibroblasts, and hepatocytes, are also susceptible to ES via voltage-opened Ca^2+^ channel [57, 80]. In particular, the piezoelectricity discovered in bone [116] and collagen [117] can also be associated to tissue regeneration. As elucidated in Figure 6(e), the piezoelectric potential of the nanofibrous web can regulate bone cell adhesion and proliferation. In this work, the surface potential of PVDF nanofibers was controlled by applying positive and negative voltages during electrospinning. Cell spreading area (Figure 6(e), I) and proliferation (Figure 6(e), II) were greatly limited when human osteoblast-like cells MG63 were cultured on the nanofibrous PVDF (+) web with stronger surface potential [102]. Nevertheless, other works demonstrated that the proliferation of preosteoblast and L929 fibroblast cell was significantly enhanced under ES exerted by nanofibrous PVDF-TrFE [85, 88]. The difference of proliferation under the piezoelectric ES of nanofibers may be attributed to the variance of ES intensity and the exploited type of cell and piezoelectric material.

In addition to proliferation, ES from piezoelectric polymeric nanofibers determines the cell differentiation toward osteocyte phenotype (Figure 6(f)) [71]. Damaraju et al. elucidated that the low-intensity voltage or streaming potential exhibited by the nanofibrous PVDF-TrFE web promoted chondrogenic differentiation while a higher one was conducive to osteogenic differentiation [6]. Furthermore, the different types of stem cells electrically stimulated by PLLA nanofibers obviously differentiated into osteogenic *in vitro*. The *in vivo* experiments revealed that it can induce desirable bone formation under the *in situ* ES of the PLLA nanofibrous web in a critical-sized calvarial defect (Figure 6(f)) [71].

Among various bone injuries, bone defects at the joint have an unsatisfied clinical demand and remain a serious challenge in orthopaedic surgery [84]. To this end, the piezoelectric nanofibrous web can be implanted into the joint part to scavenge bending energy for ES-based regeneration therapy. Fernandez-Yague et al. reported that body movement-activated PVDF-TrFE nanofibers providing *in situ* ES on the tendon brought ion channel regulation *in vitro* and mediated particular regeneration signaling pathways *in vivo* [62]. Very recently, Liu et al. introduced a therapy strategy based on exercise-driven piezoelectric ES for fascinating cartilage regeneration in rabbits. The PLLA nanofibrous web was transplanted into cartilage defect. *In situ* ES generated by the movement of the rabbit increased extracellular protein adsorption, assisted cell migration and recruitment, and induced cytokine secretion. Therefore, good cartilage repair effect can be achieved without adding additional stem cells and growth factors [84].

As compared to the well-known ES-based bone therapy, the effect of ES on mediating hepatocyte activity has been less understood. Li et al. fabricated graphene oxide (GO)/poly (3,4-ethylenedioxythiophene) (PEDOT)/Fe_3_O_4_/PAN piezoelectric nanofibers for primary hepatocyte culturing. This nanofibrous web provided *in situ* ES for enhancing hepatocyte long-distance migration and strengthening the information exchange among adjacent cells. The *in vivo* demonstration for liver injury revealed that ES can mitigate inflammation (Figure 6(g), I) and accelerate angiogenesis (Figure 6(g), II) [57].

## 5. Piezoelectric Stimulation Systems and Cell Behavior Regulation

**Figure 7:**
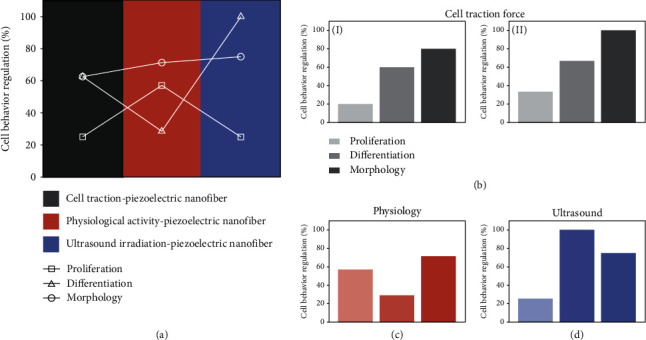
Quantitative comparisons of the electrical stimulation (ES) effects obtained using various piezoelectric systems on various cell types. (a) ES effects using various piezoelectric systems on cell proliferation, differentiation, and morphology mediation. (b) ES effect of cell traction-piezoelectric nanofiber system on (I) electrically excitable cell and (II) nonelectrically excitable cell. (c) ES effect of physiological activity-piezoelectric nanofiber system on nonelectrically excitable cell. (d) ES effect of ultrasound-piezoelectric nanofiber system on nonelectrically excitable cell.

To evaluate the ES effects using various piezoelectric systems on various cell behaviors, the quantitative comparisons are carried out on the basis of reference summary. Specifically, the ratio of cell behaviors (i.e., cell proliferation, differentiation, and morphology mediation) mediated in the specific piezoelectric system was quantitatively compared. As mentioned in Section 3.1, 3.2, and 3.3, there are three piezoelectric electrical stimulation systems, including the cell traction-piezoelectric nanofiber system (namely, cell traction system), physiological activity-piezoelectric nanofiber system (namely, physiological activity system), and ultrasound-piezoelectric nanofiber system (namely, ultrasound system) for wirelessly electrical therapy. The cell behaviors of cell proliferation, differentiation, and morphology mediation regulated with these piezoelectric systems are demonstrated in Section 4. As illustrated in Figure 7(a), almost all piezoelectric systems enable the mediation of cell morphology, and the favorable mediation effect is similar in various systems, indicating the close relation of piezoelectric stimulation on cell morphology instead of the way of mechanical force application. Interestingly, it seems that the cell traction system, physiological activity system, and ultrasound system play complementary roles in the regulation of cell proliferation and differentiation. Specifically, the cells stimulated via the cell traction system and ultrasound system feature high-level differentiation but low proliferation while those stimulated through the physiological activity system have desirable proliferation but unsatisfactory differentiation. In particular, the ultrasound system is conducive to efficient cell differentiation and morphological modulation, showing great potential in postoperative adjuvant therapy.

The ES effect of various piezoelectric systems on electrically excitable cells and nonelectrically excitable cells is further elucidated in Figures 7(b)–7(d). For electrically excitable cells in the cell traction system (Figure 7(b), I), cell morphology is the easiest behavior to be regulated, followed by proliferation and, finally, differentiation. The cell behaviors of nonelectrically excitable cells share a similar trend in the cell traction system with enhanced induction effect (Figure 7(b), II). These results reveal that the cell traction system has significant advantages in inducing cell differentiation and cell morphology mediation. In addition, nonelectrically excitable cell seems to be more susceptible to the ES supplied by the cell traction system. As nonelectrically excitable cells are commonly exploited in the physiological activity system and ultrasound system, only the effect of the piezoelectric system on the nonelectrically excitable cell is illustrated. As shown in Figure 7(c), the physiological activity system efficiently inducts the change of cell morphology. Unsimilar to the cell traction system, the regulation of cell proliferation is better than that of cell differentiation in the physiological activity system. It is worthy to note the inductive effect of cell proliferation in the physiological activity system is most fascinating among all piezoelectric systems, enabling the cell expansion in vitro via physiological activity simulation. On the contrary, the ultrasound system has an extremely remarkable advantage in inducing cell differentiation compared with the cell traction system and physiological activity system (Figure 7(d)). Nevertheless, the regulation of cell proliferation is undesirable. This feature is favorable to wirelessly induce cell differentiation *in vivo* after the implantation of the scaffold with expanded cells.

Due to the subtle ES triggered by the little to no cell traction force, the interaction of the cell and piezoelectric nanofiber may be weak, leading to the insignificant proliferation and differentiation (note that cell proliferation and differentiation are pivotal sections in tissue regeneration) in the cell traction system. Nevertheless, it is of importance in exploring the fundamental principle of the interaction between the cell and the single piezoelectric nanofiber at the cell level which may guide the efforts in the physiological activity system and ultrasound system. Obviously, the physiological activity system and ultrasound system have practical value in tissue regeneration as a result of the fascinating effect of inducing cell proliferation and differentiation, respectively. A process of regeneration therapy can be proposed on the basis, in which the cell expansion on the piezoelectric nanofibrous scaffold *in vitro* can be first achieved via physiological activity simulation, and then, these expanded cells transplanted into the body are capable of differentiating with the assistance of wireless ultrasound irradiation.

## 6. Current Challenges and Solutions

**Figure 8:**
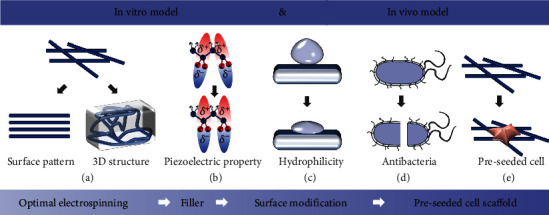
Open challenges and proposed solutions for *in vitro* and *in vivo* models: (a) surface pattern and 3D structure design; (b) piezoelectrical performance enhancement; (c) improvement of cell incubation condition; (d) antibacterial and anti-inflammatory effect; (e) cell coculturing.

Despite remarkable advantages and effectiveness of the nanofibrous piezoelectric polymer web on providing localized ES for tissue regeneration therapy, the plane structure (Figure 8(a)), relevantly low electrical output (Figure 8(b)) [118–121], poor surface condition (Figure 8(c)) [122, 123], and possible infection during transplantation (Figure 8(d)) can limit the application scope and electrical signal transmittance of the piezoelectric nanofiber web. Hence, optimized strategies are urgently required to create an ideal electroactive platform as the *in vitro* model. Furthermore, the *in vivo* model calls for an additional physicochemical property to cope with possible problems in scaffold transplantation (e.g., inflammation and bacterial infection) and meet the specific demand of tissue growth (Figure 8(e)) and drug synergistic therapy. To this end, strategies of optimizing electrospinning parameters, one-/two-dimensional hybrid composites, surface modification, and selected cell for the preseeded cell-scaffold system are summarized to propose a desirable electroactive scaffold for *in vitro* research and *in vivo* clinical application (Figure 8).

### 6.1. Electrospinning Parameters

**Figure 9:**
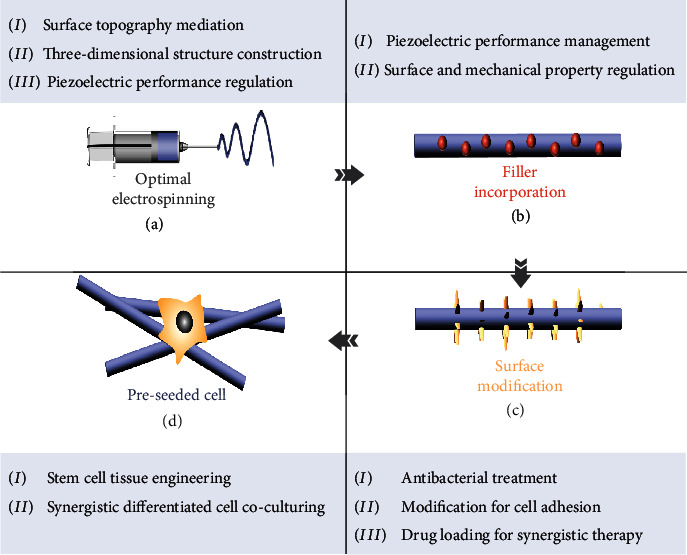
Key aspects for fostering effective stimulation. (a) Adjusting of electrospinning parameters for controlling physical structure and piezoelectric performance. (b) Filler incorporation to induce higher piezoelectric crystal and regulate physical property. (c) Surface modification for favorable cell growth condition and drug synergistic therapy. (d) Cocultured with stem cell or helper cell toward improved cell development and tissue regeneration.

The nanofibrous polymer scaffold featuring piezoelectric performance can be handily achieved via one-step electrospinning technology. Regulating the spinning and environment conditions (solution concentration, applied electric field, collector RH, etc.) is desirable for modulating the physical structure and piezoelectric stimulation of this scaffold which plays a dominant role in regulating cell growth and development (Figure 9(a)). Strategies for the modulation of structure and piezoelectric performance via controlling electrospinning parameters and its corresponding cell response are summarized in Table 2.

#### 6.1.1. Surface Topography Mediation

Various surface patterns of the piezoelectric fibrous scaffold open the distinct mechanotransduction signal pathway, thus synergistically regulating cell activities with the electrotransduction signal pathway. The collector with topography mediation allows the tunable control of the fibrous structure for fabricating a specific patterned scaffold [124]. A random, aligned, or radially aligned fibrous scaffold could be realized through the collector base in combination with copper wire and cellophane tape [125]. Additionally, a rotating drum collector with high speed is an efficient way to obtain aligned nanofibers which is favorable for directional cell growth [109, 126]. Apart from one single-patterned scaffold, a multipattern could also be integrated into the same fibrous scaffold through directly taping the conductive templates with multiple microstructures. A gradient topography enables distinctive mechanical properties which is favorable to mimic the natural tissues [127].

#### 6.1.2. Three-Dimensional Structure

For the tissues with the possibility of suffering bulk damage, like bone, the 3D scaffold is necessary to support cell growth and proliferation [6], and an ideal porous structure benefits cell infiltration [128, 129]. Despite the porous structure being readily obtained [130–132], the construction of the three-dimensional nanofibrous structure remains a challenge [133]. Kim et al. demonstrated that the 3D PVDF nanofibrous structure could be realized under a humid condition with RH exceeding 90% [134]. It was believed that high RH can lead to a residual solvent on the fiber surface, the solvent residue charged by electric field-endowed adjacent fibers with charge repulsions which brought out a cotton-like 3D structure [135]. In addition to RH, a new configuration of electrospinning-applied voltage was also presented for 3D design. In this work, an electrode with an applied voltage of 25-28 kV was still connected to the needle; another electrode with a power supply of 2-10 kV is connected to the stainless steel plate, instead of grounding [6].

#### 6.1.3. Piezoelectric Performance Regulation

Lee et al. introduced Equations (1) and (2) to evaluate the sensing and energy harvesting capabilities of piezoelectric material (FOM, referring to figures of merit) [136].

(1) $$\begin{array}{c} {\left(\text{FOM}\right)}_{\text{voltage}}=g=\frac{d}{\varepsilon }, \end{array}$$

(2) $$\begin{array}{c} {\left(\text{FOM}\right)}_{\text{power}}=d\times g=\frac{d^{2}}{\varepsilon }, \end{array}$$

where *d* is the piezoelectric charge coefficient, *g* is the piezoelectric voltage coefficient, and *ε* is the dielectric constant. These equations indicate that the enhancement of overall piezoelectric performance should focus on either increasing *d* or decreasing *ε*. Generally, the strategy of enhancing *d* is to increase the electroactive phase of the piezoelectric material [137, 138] which is handily achieved by adjusting electrospinning parameters, such as electric field strength [65, 139], solution concentration [140], thermal treatment [113], and drum collector rotating speed [141]. For example, Tai et al. demonstrated that the nanofibrous PLLA web scaffold with finer fiber diameter was obtained via reducing the concentration of spinning solution. The nanofibrous web with finer fiber diameter displayed a higher piezoelectric charge coefficient [113]. In addition to inducing the formation of piezoelectric crystals, morphology engineering is another factor enabling higher piezoelectric output for decreasing *ε* [142, 143]. To be more specific, the porous nanofiber is conducive to a smaller *ε* value for enhanced piezoelectric performance, despite having a poor electroactive phase [136].

### 6.2. Fillers

The improvement of piezoelectric property via adjusting the parameters of electrospinning is limited due to the semicrystalline structure of most piezoelectric polymers. Compared with the piezoelectric polymer, inorganic piezoelectric materials feature high piezoelectric performance, and the interaction between fillers and the polymer matrix benefits electroactive phase formation [86]. For this reason, a series of nanoparticles or nanowires of the piezoelectric crystal [144], piezoelectric ceramics [40, 145], polymers [146], other fillers [147–149], or their blends [150] have been incorporated into the electrospun fibrous polymer matrix to improve piezoelectricity while simultaneously endowing the scaffold with additional functions (Figure 9(b)). The strategies of improving the piezoelectric and physical performance via incorporating fillers into the polymer matrix are summarized in Table 3.

#### 6.2.1. Piezoelectric Performance Management

By the virtue of the piezoelectric property, ZnO nanoparticles and nanowires have been extensively utilized and incorporated to strengthen the piezoelectric property of the fibrous piezoelectric scaffold [144, 151]. The incorporated ZnO fillers also enable the inhibition of bacterial growth [152] and inflammation [153], which is beneficial to more angiogenesis compared with pristine nanofibers. In comparison with the piezoelectric crystal, the advantages of the high piezoelectric property and excellent chemical stability make piezoelectric ceramics, such as BaTiO_3_, a desirable candidate as filler in the fibrous piezoelectric scaffold [154, 155]. On the other hand, nonpiezoelectric materials of MWCNTs [156], IL [157, 158], and DA [159] have been introduced to induce a more piezoelectric phase owing to the dipolar interaction of the filler and piezoelectric polymer [34, 86, 159]. In order to apply a controllable electrical stimulation, magnetic nanoparticles serving as the component for magnetic actuation have also been employed, so the piezoelectric polymer can act as both a magnetically steerable scaffold and an acoustically responsive cell electrostimulation platform [54, 160, 161]. In spite of many merits possessed by filler incorporation, it is worthy to note that excessive addition of fillers would have an adverse effect on the cell viability and proliferation [160, 162].

Notably, the distribution uniformity [36] and density [35] of filler in the piezoelectric polymer matrix have a significant influence on its piezoelectric properties. Furthermore, the considerable mismatch of mechanical moduli and poor interfacial adhesion between rigid fillers and the flexible piezopolymer matrix leads to a barrier for combined utilization, which extremely hinders the ability of stress transfer and thus largely restrains the electromechanical coupling efficiency [32]. To tackle this, surface modulation on the filler is generally selected to bridge the interaction between fillers and polymer chains [40, 163, 164].

#### 6.2.2. Surface and Mechanical Property Regulation

Fillers embedded into the fibrous matrix not only induce enhanced piezoelectric performance but also play a crucial role in improving the surface and mechanical property of the matrix. For example, composite TiO_2_/HA fillers were incorporated into a PMMA/PVDF matrix, and the nanofibrous PVDF/PMMA/HA/TiO_2_ composite exhibited a hydrophilic surface with a contact angle of 39° [150]. An *in vitro* test illustrated that this nanofibrous web was desirable for the adhesion of cardiomyocytes.

In addition to excellent surface property for cell adhesion, the basic mechanical properties of the piezoelectric scaffold are necessary for complex using environment *in vitro* and in vivo. He et al. illustrated that a 2 wt% organosilicate incorporation in the nanofibrous PVDF-TrFE web can lead to significant improvements in strength and toughness by about 103% and 97%, respectively [165]. Moreover, the incorporation of PU has been verified to improve the elasticity of the nanofibrous PVDF web, satisfying the requirement of mechanical compatibility towards skin tissue [166].

### 6.3. Surface Modification

The tissue scaffold is incorporated to support and guide cell development; the surface property of the scaffold is crucial in the communication between the cell and scaffold or among cells. Despite filler embedment partially improving the surface property, this enhancement can be limited due to the encompassed polymer matrix. Hence, direct surface modification is implemented on the scaffold for favorable cell growth and the synergistic therapy of chemical drug and ES-based therapy (Figure 9(c)). The formulated schemes are listed in Table 4.

#### 6.3.1. Antibacterial Treatment

When a scaffold is transplanted *in vivo*, the possible bacterial infection of the wound site can sometimes influence the effect of tissue regeneration. Thus, a scaffold with antibacterial property is necessary for transplantation. Badaraev et al. prepared a PVDF-TeFE nanofibrous web with an antibacterial Cu coating for the tissue regeneration of the oral mucosa [167]. *In vivo* studies demonstrated that this piezoelectric nanofibrous web with an antibacterial coating contributed to the regeneration of oral mucosa to the greatest extent. Moreover, antibacterial HA was also presented for the surface antibacterial modification of the piezoelectric PVDF scaffold through the electrodeposition method. The PVDF/HA scaffold showed a 99.8% efficiency against Pseudomonas aeruginosa bacteria while containing the highest cell viability, total protein, and alkaline phosphatase activity of MG63 cells by 7 days [168].

#### 6.3.2. Modification for Cell Adhesion

After seeding cells on the scaffold, various adhesion proteins on the cell membrane first contact the scaffold and sense the physicochemical signal of the scaffold surface. The binding degree between the adhesive protein and the scaffold determines cell adhesion which is the fundamental section for subsequent cell activities. Conventionally, the surface wettability of the scaffold plays a significant role in the cell adhesive process [169]. Nevertheless, commonly utilized piezoelectric polymer scaffolds are hydrophobic, which hinders sufficient cell expansion and penetration.

Thus, plasma treatment was introduced to endow the hydrophobic scaffold with the hydrophilic surface [80, 123]. Despite plasma treatment easily realizing the hydrophilic and rough surface which benefits cell adhesion and spreading [80], it is not the most ideal strategy for durable surface treatment due to transient effect and tedious device. Deposition of hydrophilic compounds is another common strategy for achieving the hydrophilic surface [170, 171]. For instance, the PHB scaffold could be directly immersed into the hydrophilic chemical diazonium of ADT-(COOH)_2_ for surface hydrophilic treatment [172]. It was demonstrated that the density of osteoblastic cells increased on the surface of the ADT(COOH)_2_-treated scaffold in comparison to that on the pristine one. It is interesting to note that surface charges are desirable for the even dispersion of the deposited layer [118]. Additionally, surface hydrophilicity could also be incorporated via coaxial electrospinning where the hydrophilic material is displayed in the shell component while the piezopolymer is distributed in the core component. Departing from improving the surface property for protein adhesion, directly introducing adhesive protein and signal molecules have also been proposed [173]. The human embryonic stem cells cultured on a nanofibrous PVDF web with vitronectin-derived peptide-mussel adhesive protein fusion coating were stably expanded for more than 10 passages, maintaining the expression of pluripotency markers and genomic integrity [174].

#### 6.3.3. Drug Loading

Compared with the single function of antibiosis, hydrophilicity, and signal factors achieved by surface modification, drug synergistic therapy can be more favorable owing to the integration of multiple functions via drug loading and the controllability through external stimuli. A multifunctional scaffold integrating with hydrophilicity, antibacterial feature, and bioactive molecules has been successfully fabricated via drug carrying. In this work, ultrasound-induced surface charges promoted the *in situ* synthesis of CaCO_3_ which endowed the piezoelectric scaffold with hydrophilicity and increased the efficiency of drug loading [171]. The CaCO_3_-modified PHB scaffold containing both glycopeptide antibiotic vancomycin (VCM) and enzyme alkaline phosphatase (ALP) molecules exhibited the highest cell density while the VCM-loaded one had a remarkable antibacterial effect against gram-positive bacteria Staphylococcus aureus. Additionally, Timin et al. illustrated a multifunctional nanofibrous piezoelectric web integrating with antibiosis and signal factors through capsule decoration. The antibiosis and signal factors encapsulated into the capsule could be controllably released via external stimuli, e.g., ultrasound, laser radiation, and enzyme treatment [175].

### 6.4. Cell-Scaffold System

For a successful tissue regeneration, the cell, scaffold, and signaling molecules are three essential components [175]. As discussed above, various strategies, i.e., electrospinning parameters, surface modification, and fillers, have been proposed to endow the nanofibrous piezoelectric scaffold with multifunctions simulating the physical, chemical, and electrical stimulations of ECM. Combining proper cells with this scaffold is a promising strategy for better therapy (Figure 9(d)). The works selecting cells for enhanced tissue regeneration are listed in Table 5.

#### 6.4.1. Stem Cell

Significant advantages of the stem cell make it a promising candidate for the scaffold-cell system. Adult stem cells, especially MSCs, have been extensively used for tissue engineering purposes due to easy autotransplantation [114]. For example, Augustine et al. elucidated that better regeneration effect with preseeded MSCs was achieved under the *in situ* ES of the piezoelectric nanofibrous web. In this work, a highly branched and increasing number of vasculature were formed on nanofibrous ZnO/PVDF-TrFE web preseeding with MSCs, compared with that on the pure ZnO/PVDF-TrFE nanofibrous web [162]. However, there are great limitations in the application of MSCs, including the downtrend in cell function over time [176, 177] and tough isolation from mature tissues [178]. Therefore, hiPSCs are introduced to deal with these limitations due to no cell lineage limitations and the capability of reprogramed and reverted mature cells to pluripotent stem cells with the assistance of four transcription factors (i.e., Oct4, Sox2, Klf4, and c-Myc) [179]. Azadian et al. demonstrated that the directly seeded hiPSCs on the nanofibrous PVDF/PVA/GO web differentiated to osteoblasts, indicating a favorable scaffold-stem cell system for bone regeneration therapy [178].

#### 6.4.2. Differentiated Cell

Alternatively, other types of cells have also been selected according to specific tissue for the synergistic treatment with ES therapy. SCs play a significant role in axon regeneration of the peripheral nerve. Thus, Lee et al. prefilled SCs into nanofibrous PVDF-TrFE conduits. This piezoelectric nanofibrous conduit with prefilled SCs induced enhanced noradrenergic axon regeneration in the transection of the spinal cord [114]. Furthermore, SCs were also cocultured with DRG neurons on the aligned nanofibrous PVDF-TrFE web. It was demonstrated the SC/DRG/PVDF-TrFE system promoted longer neurite extension and the formation of myelin around DRG neurites [173].

## 7. Conclusions and Perspective

Integrating mechanical stretching with spontaneously electrical polarization, electrospinning technology has attracted unprecedented research enthusiasm in one-step fabricating fibrous polymers with piezoelectric properties. This fibrous piezoelectric web can mimic the piezoelectric collagen fibers in ECM, acting as a medium of bioelectric signal transmission and communication between cells. Moreover, the piezoelectric nanofiber unit endows the electroactive scaffold with great sensitivity for scavenging subtle force at the cell level. The combination of electrospun piezoelectric polymer nanofiber and mechanical loading, e.g., cell traction, organism motion, and ultrasound irradiation, proposes a paradigm for *in vitro*/*in vivo* electrical stimulation application in a noninvasive manner. Extensive works have demonstrated that piezoelectric electrical stimulation leads to Ca^2+^ influx which modulates cell morphology, proliferation, and differentiation via regulating gene transcription and cell polarization. Understanding the interaction of piezoelectric electrical stimulation and cell activities leads to numerous accomplishments on the regeneration therapy in electrically excitable tissue (neural and muscle tissue) and nonelectrically excitable tissue (bone and hepatocyte tissue). The further quantitative analysis demonstrates that piezoelectric ES supplied via the cell traction-piezoelectric nanofiber system, physiological activity-piezoelectric nanofiber system, and ultrasound-piezoelectric nanofiber system effectively mediate cell morphology. In addition, there are complementary roles in various piezoelectric systems over the regulation of cell proliferation and differentiation. In particular, the physiological activity system and ultrasound system have the fascinating advantage of inducing cell proliferation and differentiation, respectively. A combined process of regeneration therapy can be proposed on the basis in which the simulation of the physiological activity system *in vitro* enables the cell expansion while wireless ultrasound irradiation allows the differentiation of the expanded cells transplanted into the body.

Nevertheless, a system model mimicking ECM for *in vitro* research and *in vivo* application is still missing, due to the intrinsic defects of piezoelectric polymers as tissue scaffolds and possible crisis in transplantation. To this end, the optimization of the piezoelectric polymer scaffold and its clinical translation must be guaranteed via a series of steps: (i) enhancement of piezoelectric output, (ii) surface pattern and 3D structure design, (iii) antibacterial treatment, (iv) improvement of surface property for cell adhesion, and (v) stem cell therapy and preseeded with assisted growth cell. Therein, the remarkable electrical stimulation and effective electrical signal transmittance make (i) to (iv) inevitable for proposing an *in vitro* model. For the *in vivo* model, all aspects need to be mentioned. For example, the surface pattern is beneficial for fast tissue formation while the 3D scaffold is necessary for the bulk defects, like the spinal cord. Overall, some of these pieces have reached a relatively high level of maturation; others are still puzzles and need to be further explored for clinical purpose. Hopefully, future efforts will bring out more desirable results, therefore transforming this conception into practical clinical therapy.

## Figures and Tables

**Table 1:** Application strategies of electrical stimulation and the corresponding cell response.

Piezoelectric polymer	Electrical stimulation	Application method of electrical stimulation	Cell response	Refs.
PAN	1.41 mV (two nanofibers)	Cell traction	Viability, differentiation, and maintain functional expression	[57]
PLLA	~3 mV (single fiber)	Cell traction	Adhesion and differentiation	[65]
PVDF	0.73-133 mV	Cell traction	Differentiation	[56]
PVDF	Surface charges	Cell traction	Adhesion, expansion, and penetration	[80]
PVDF	Surface charges	Cell traction	Expansion and proliferation	[180]
PVDF	Surface charges	Cell traction	Adhesion, expansion, and differentiation	[125]
PVDF-TrFE	Surface charges	Cell traction	Neurite extension	[109]
PVDF-TrFE	7 × 10^−5^ V	Cardiomyocyte beating	Differentiation and maturation	[81]
PVDF/DA	~40/2.5 mV	Breathe/blood flow	—	[159]
PVDF/ZnO/rGO	~3.9 V	Cardiac motions	—	[86]
PVDF/PDA-PAAm	0.1-0.5 V	Motion of mouse	Proliferation and migration	[54]
PLLA	~2 V	Exercise of rabbit	Migration and differentiation	[84]
PVDF	Surface charges	Mechanical stretching (1 Hz/10 mm strain)	Proliferation	[152]
PVDF	Surface charges	Bidirectional cyclic bending	Viability	[154]
PVDF-TrFE	1.20 mV/mm; 1 V/mm	Cyclic compression (1 Hz/10% deformation)	Differentiation	[6]
	61.1 ± 1.5 *μ*V; 25.2 ± 2.5 *μ*V			
PVDF/PU	Surface charges	Intermittent deformation (0.5 Hz/8% deformation)	Adhesion and migration	[181]
PVDF-TrFE	12 nA/14 nA (current)	Custom-made speakers (2 Hz, 4 V)	Proliferation	[88]
	1.2 V/-1.7 V(voltage)			
PVDF-TrFE	0.3 V	Hydroacoustic waves	Differentiation	[112]
PVDF	Surface charges	Magnetic field	Proliferation and differentiation	[52]
PLLA	~ 70 mV	Ultrasound (40 kHz)	Differentiation	[71]
PLLA	0.5 V	Ultrasound (300 W)	Stemness maintenance and proliferation	[98]
PVDF/FeOOH	Surface charges	Ultrasound (400 W)	Differentiation	[50]

Abbreviation: PAN: polyacrylonitrile; PLLA: poly(L-lactic acid); DA: dopamine; ZnO: zinc oxide; rGO: reduced graphene oxide; PDA-PAAm: polydopamine-polyacrylamide; PVDF-TrFE: poly(vinylidene fluoride-trifluoroethylene); PU: polyurethane.

**Table 2:** Modulation of structure and piezoelectric performance via controlling electrospinning parameters and its corresponding cell response.

Polymer	Electrospinning parameters	Supplemental effect	Cell response	Refs.
PVDF	Collector configuration	Aligned or radially aligned pattern	Alignment, proliferation, and differentiation	[125]
PVDF–TrFE	Drum collector speed	Aligned pattern	Directed outgrowth	[109]
PVDF–TrFE	Drum collector speed	Aligned pattern	Directed outgrowth	[81]
PVDF	Drum collector speed	Aligned pattern	Directed outgrowth	[56]
PVDF	Collector configuration	Honeycomb-like to randomly oriented pattern	—	[127]
PVDF–TrFE	Two electrodes with different applied voltage	3D nanofibrous structure	Differentiation	[6]
PVDF	Ambient humidity	3D nanofibrous structure	—	[134]
PVDF–TrFE	Ambient humidity	Piezoelectric performance	—	[136]
PLLA	Electric field strength	Gradient piezoelectricity	Differentiation	[65]
PVDF	Applied voltage	Piezoelectric performance	Alkaline phosphatase activity and early mineralization	[139]
PLLA	Thermal treatment/spinning solution concentration	Piezoelectric performance	Differentiation	[113]

**Table 3:** Performance enhancement via the incorporation of fillers and its corresponding cell response.

Polymer	Filler	Supplemental effect	Cell response	Refs.
PVDF, PHBV	HA, SiHA nanoparticles	Piezoelectric performance	Adhesion and differentiation	[147, 149]
PVDF	Au nanoparticles	Piezoelectric performance	Viability and adhesion	[148]
PVDF-TrFE, PVDF, PU	ZnO nanorods, ZnO nanoparticles	Piezoelectric performance, anti-inflammatory, and antimicrobial property	Viability and proliferation	[151–153, 182]
PVDF-TrFE	TiO_2_ nanowires	Strength	Adhesion and proliferation	[183]
PVDF, PCL	BaTiO_3_ nanoparticles	Piezoelectric performance	Viability and proliferation	[154, 155, 166]
PVDF	PDA/BaTiO_3_ nanoparticles	Piezoelectric performance	—	[32]
PVDF-TrFE	PMMA/BaTiO_3_ nanowires	Piezoelectric performance	—	[40]
PHB	MWCNTs	Piezoelectric performance	—	[156]
PVDF	POSS–EGCG	Piezoelectric performance	Proliferation and differentiation	[184]
PHBV, PVDF	CoFe_2_O_4_ nanoparticles	Strength and degradation	Viability	[160, 161]
PVDF	GO/CoFe_2_O_4_ nanoparticles	—	Proliferation and differentiation	[52]
PVDF	Organosilicate nanoplates	Strength and toughness	—	[165]
PVDF	PU	Strength and elongation	Adhesion and migration	[181]
PVDF	PPy, PANI	—	Viability	[185]
CS	PEDOT	—	Proliferation and differentiation	[186]
PVDF-PVA	GO nanosheets	Strength	Proliferation and differentiation	[178]
PVDF	CsPbBr_3_ nanoparticles	Piezoelectric performance	—	[33]
PVDF	Ionic liquid (IL)	Piezoelectricity and strength	Proliferation	[157, 158]

Abbreviation: PHBV: poly(hydroxybutyrate-valerate); HA: hydroxyapatite; SiHA: silicate-containing hydroxyapatite; TiO_2_: titanium dioxide; PCL: polycaprolactone; PDA: polydopamine; PMMA: polymethyl methacrylate; PHB: polyhydroxybutyrate; MWCNTs: multiwalled carbon nanotubes; POSS–EGCG: poly(vinylidene fluoride) composite nanofibers containing polyhedral oligomeric silsesquioxane; PVA: polyvinyl alcohol; GO: graphene oxide; PPy: polypyrrole; PANI: polyaniline; CS: chitosan; PEDOT: poly (3,4-ethylenedioxythiophene).

**Table 4:** Surface property modulated via surface modification and the corresponding cell response.

Polymer	Decorated material	Methods	Supplemental effect	Cell response	Refs.
PVDF	Oxygen plasma	Plasma treatment	Hydrophilicity	Cell adhesion, expansion, and penetration	[80]
PLLA	Oxygen and argon plasma	Plasma treatment	Hydrophilicity	Viability	[123]
PHB	ZnO nanoparticles	Hydrothermal deposition	Antibacteria, hydrophilicity	—	[170]
PVDF	ZnO nanorods	Hydrothermal method	Piezoelectric performance	—	[144]
PVDF-TeFE	Cu nanoparticles	Magnetron sputtering	Antibacteria	Wound healing	[167]
PVDF	HA nanoparticles	Electrodeposition	Antibacteria	Cell viability, total protein, and alkaline phosphatase activity	[168]
PHB and PHBV	CaCO_3_	Chemical deposition	Hydrophilicity	Adhesion and proliferation	[118]
PHB	ADT(COOH)_2_	Dip coating	Hydrophilicity	Expansion and proliferation	[172]
PVDF	Vitronectin-derived peptide	Surface coating	—	Long-term maintenance and differentiation	[174]
PVDF–TrFE	Matrigel	Dip coating	—	Extension and myelination	[173]
PVDF	PDA-PAAm hydrogel	Surface adherent hydrogel	—	Proliferation, migration, and expressions of crucial growth factors	[45]
PHB, PHB-PANi	Capsules	Oscillation attachment	Antibacteria	Differentiation	[175]

Abbreviation: ADT(COOH)_2_: 3,4-dicarboxybenzenediazonium tosylate.

**Table 5:** Selected cell for cell-scaffold system and its regeneration effect under *in situ* electrical stimulation of piezoelectric nanofibers.

Polymer	Preseeded cell	Cell response/regeneration effect	Refs.
PVDF-TrFE/ZnO	MSCs	Angiogenesis	[162]
PVDF-TrFE	hiPSCs	Cardiac differentiation and maturation	[81]
PVDF-TrFE	SCs	Spinal cord transection repair	[114]
PVDF-TrFE	SCs	Neurite extension and myelination	[173]
PLLA	Adipose stem cells and MSCs	Osteogenic differentiation	[71]

Abbreviation: MSCs: mesenchymal stem cells; hiPSCs: human induced pluripotent stem cells; SCs: Schwann cells.
